# Optical coherence tomography angiography reveals abnormal retinal vascular density and perfusion in patients with X-linked adrenoleukodystrophy: a cross-sectional study

**DOI:** 10.1186/s13023-024-03499-x

**Published:** 2025-01-13

**Authors:** Lujie Zhang, Yongqiu Yu, Ting Liu, Chongyi Li, Liang Tan, Shuiqian Wen

**Affiliations:** 1 Department of Ophthalmology, Daping Hospital, Chongqing, China; 2 Department of Neurosurgery, Southwest Hospital, Chongqing, China

**Keywords:** X-linked adrenoleukodystrophy, Optical coherence tomography angiography, Retinal vascular, Neurodegeneration

## Abstract

**Purpose:**

X-linked adrenoleukodystrophy (XALD) can affect the eyes. Existing therapies are hampered by early quantitative examination methods. This study used an optical coherence tomography angiography system (OCTA) to investigate retinal microvascular density and perfusion in XALD patients.

**Methods:**

Fifty-two patients and 47 age-matched controls were included in this cross-sectional study. The patients were divided into three groups (symptomatic, less symptomatic, and controls). We compared the foveal avascular zone area, vascular density and perfusion area at the superficial vascular complex (SVC) and deep vascular complex (DVC) of the peripapillary and macular between the groups. We correlated these measurements with scale scores.

**Results:**

Compared with the controls, the symptomatic group had significantly lower vascular density in the superior nasal sector of the peripapillary SVC (MD − 4.940884; 95% CI − 9.655061 to − 0.226707; p = 0.036), lower vascular density (MD − 4.259225; 95% CI − 8.248627  to  − 0.269823; p = 0.032) and lower perfusion area (MD − 0.180304; 95% CI − 0.337135  to  − 0.023472; p = 0.018) in the peripheral ring superior quadrant of the macular SVC. Compared with the less symptomatic group, the symptomatic group exhibited a significantly lower vascular density (MD − 5.635483; 95% CI − 10.450009  to  − 0.820957; p = 0.015) and perfusion area (MD − 0.063351; 95% CI − 0.116611  to  − 0.010091; p = 0.013) in the superior nasal sector of the peripapillary SVC; lower vascular density (MD − 4.817846; 95% CI − 8.924294  to  − 0.711399; p = 0.015) and perfusion area (MD − 0.202707; 95% CI − 0.369499  to  − 0.035915; p = 0.011) in the peripheral ring superior quadrant of the macular SVC; and greater vascular density (MD 7.209401; 95% CI 0.818716–13.600086; p = 0.021) and perfusion area (MD 0.047320; 95% CI 0.001685–0.092956; p = 0.039) in the inferior nasal sector of the peripapillary DVC. Among the 52 patients, the expanded disability status score (EDSS) was moderately negatively correlated with the vascular density (p = 0.001) and perfusion area (p = 0.002) in the peripheral ring superior quadrant of the macular SVC.

**Conclusion:**

Changes in retinal vascular density and perfusion exist in XALD patients and are correlated with disease severity. OCTA has the potential to monitor the progression of XALD.

**Supplementary Information:**

The online version contains supplementary material available at 10.1186/s13023-024-03499-x.

## Introduction

X-linked adrenoleukodystrophy (XALD) is a rare neurodegenerative disease caused by mutations in the Xq28 gene on the X chromosome [23]. Mutations in this gene render its encoded product, ALDP, a peroxisomal ABC half-transporter, unstable [27]. ALDP is involved in the process of transporting CoA-activated very long-chain fatty acids (VLCFAs) into peroxisomes. ABCD1 gene mutations affect the above process, causing damage to the beta-oxidation of VLCFAs and ultimately leading to the accumulation of VLCFAs in the cytoplasm, which in turn leads to the accumulation of VLCFAs in the body [31, 46], especially in the white matter of the cerebrum, spinal cord and adrenal cortex, leading to impaired function of the above organs [11]. Therefore, XALD can also be considered a metabolic disease.

XALD can be divided into several different types, including adrenocortical insufficiency, cerebral ALD (CALD) and adrenomyeloneuropathy (AMN) [11]. Adrenocortical insufficiency is the most common clinical phenotype of male patients with XALD. At least 70% of male patients experience such symptoms, which are mainly systemic symptoms caused by insufficient secretion of glucocorticoids [8, 14]. In male patients, AMN is also not uncommon and usually develops in adulthood [48]. AMN manifests with symptoms such as gradually progressive spastic paraparesis, sensory ataxia, sphincter dysfunction, leg pain and impotence [26], which are caused primarily by axonal degeneration [44]. AMN may also lead to cerebral involvement [43]. Among these phenotypes, the most severe and lethal is CALD, which usually appears in childhood and is characterized by an early decline in attention and cognitive function [26, 35]. It first manifests as a decline in academic performance, accompanied by defects in visuospatial and visuomotor functions. Loss of visual acuity, homonymous hemianopsia and visual agnosia may also be caused by impairments in the cerebral cortex [11, 40]. The disease progresses very quickly in patients with CALD. It usually only takes two to four years from the onset of symptoms to general disability or even death [11]. Cerebral lesions are caused by inflammatory demyelination of the white matter of the brain. Histopathological examination revealed large-scale demyelination in the cerebral cortex, accompanied by infiltration of astrocytes and macrophages [21, 32].

Currently, bone marrow transplantation and allogeneic haematopoietic stem cell transplantation are considered to have certain therapeutic effects on XALD. These methods have been proven to successfully reduce VLCFA levels, alleviate oxidative damage and delay the progression of clinical symptoms in CALD patients, but they cannot prevent AMN [16, 33, 45]. Several new treatments have emerged in recent years, such as Lenti-D gene therapy [9]. However, these treatments need to be carried out at an early stage when there is only slight cerebral demyelination or even before cerebral lesions occur [36]. If the above treatments are carried out when the Loes score is ≥ 10 or neurological dysfunction occurs, patients have a very poor prognosis [24]. However, early diagnosis is often difficult because of atypical early symptoms. As of 2016, the Recommended Uniform Screening Panel (RUSP) recommended adding XALD to state newborn screening programs. However, screening programs rely mainly on invasive examinations [48]. Clinical findings indicate that the initial symptoms of XALD patients can include strabismus (39.3%) and vision loss (21.4%) [34]. Studies have shown that the retinal nerve fibre layer and ganglion cell layer become thinner in XALD and are related to disease severity [2, 3, 42]. Moreover, cognitive decline and neuronal damage are associated with changes in retinal and choroidal blood vessel density [6, 18, 30, 47]. The purpose of this study was to use an optical coherence tomography angiography system (OCTA) to study retinal microvascular density and perfusion in patients with XALD, explore its correlation with disease severity and identify potential biological markers to monitor the progression of XALD.

## Materials and methods

This study was approved by the Bioethics Committee of Daping Hospital ((2023) No. 90) and was registered with China Clinical Trials with the registration number ChiCTR2400081141. All participants or their legal guardians signed informed consent forms.

### Study design and participants

This was a cross-sectional study in which all patients were recruited from June 2022 to February 2024 at Southwest Hospital in Chongqing, China. Patients aged between 8 and 75 years and diagnosed with XALD on the basis of clinical manifestations, laboratory tests and genetic testing were eligible to participate in the study. Moreover, we excluded patients who were unable to cooperate with the examination due to severe intellectual decline, and who had a history of intraocular surgery or other diseases that interfered with the assessment of neurological function. Healthy people were openly recruited through advertisements. All the subjects underwent relevant examinations at the Department of Ophthalmology, Daping Hospital, Chongqing, China, including assessment of neurological function and ocular examination. The ocular examination included visual acuity (Snellen acuity chart), intraocular pressure measurement with a noncontact tonometer, slit lamp biomicroscopy and fundus photography. Through ocular examination, we excluded participants with retinopathy caused by hypertension, diabetes, and vascular disease defined by the OSCAR-IB criteria [39], OCTA signal quality lower than 7 or obvious artefacts, high myopia (> 6 diopters) with fundus changes, intraocular pressure > 21 mmHg with visual field damage and severe refractive media opacity that interferes with imaging quality.

### Nervous system function assessment

The assessment of participants’ neurological function was performed via the Expanded Disability Status Score (EDSS) [17] and the Severity Scoring System for Progressive Myelopathy (SSPROM) [5] to assess the severity of their disease. The EDSS scores range from 0 to 10, with higher scores indicating more severe disability [17]. SSPROM scores range from 0 to 100, with lower scores indicating greater spinal cord injury [5]. Neurological function assessment was performed on the same day as OCTA imaging. Through medical history inquiry and neurological function assessment, patients with typical symptoms and signs of XALD or with an EDSS score greater than 4 and an SSPROM score less than 90 were classified into the symptomatic group, and the remaining patients were classified into the less symptomatic group.

### OCTA

**Fig. 1:**
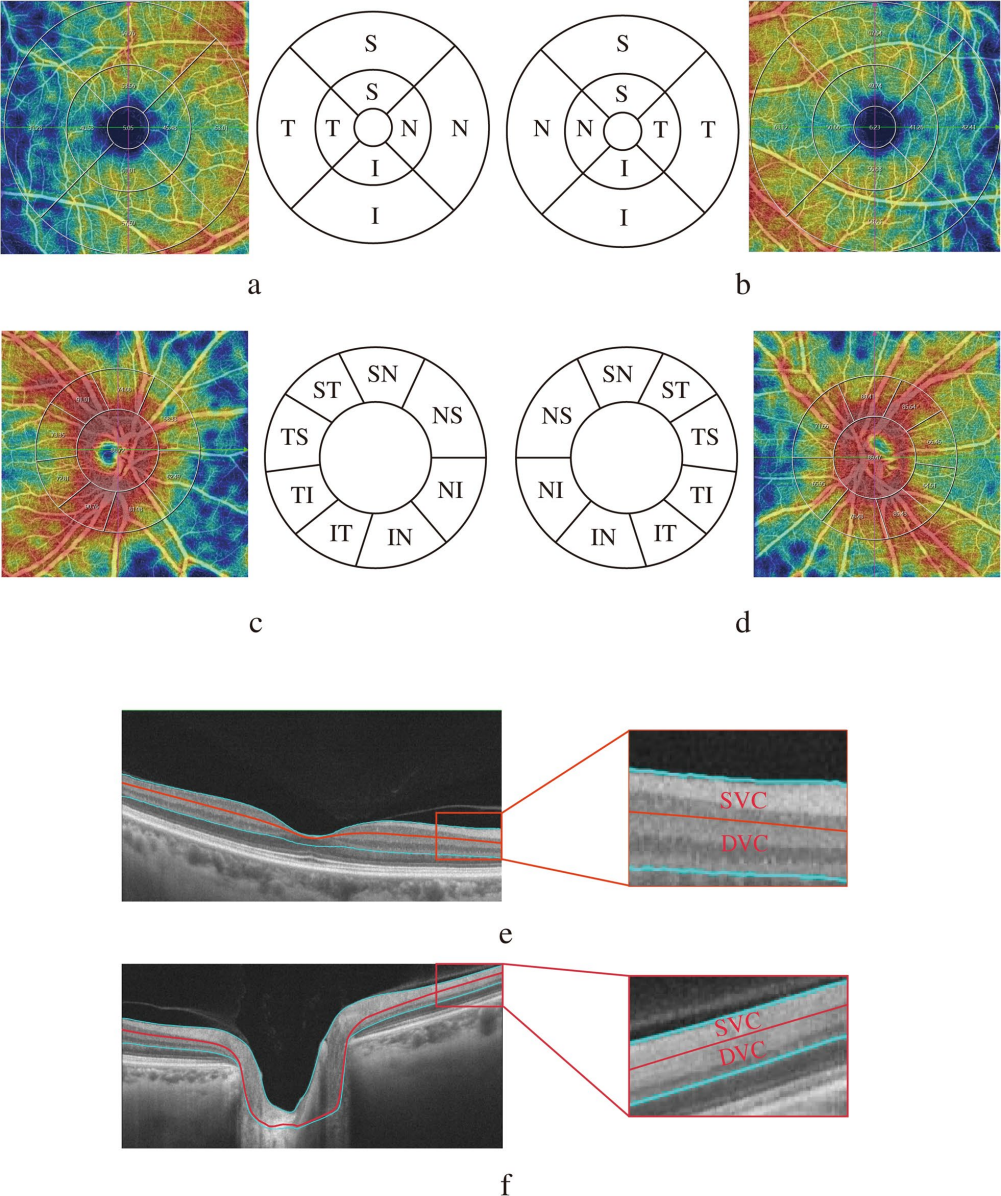
Macular and peripapillary partitioning and stratification methods. **a** The right eye macular is segmented for the ETDRS segmentation network. The peripheral ring and pericentral ring are divided into four quadrants: S, I, N and T. The red and green lines are reference auxiliary lines. **b** Segmenting the left eye macular for the ETDRS segmentation network. **c** The right eye peripapillary region is segmented for the ONH segmentation network, and the surrounding area of the peripapillary region is divided into eight sectors: NS, NI, IN, IT, TI, TS, ST and SN. **d** Segmenting the left eye optic disc for the ONH segmentation network. **e** The macular is divided into superficial and deep layers: SVC: superficial vascular complex; DVC: deep vascular complex. **f** The peripapillary layer is divided into superficial and deep layers

**Fig. 2:**
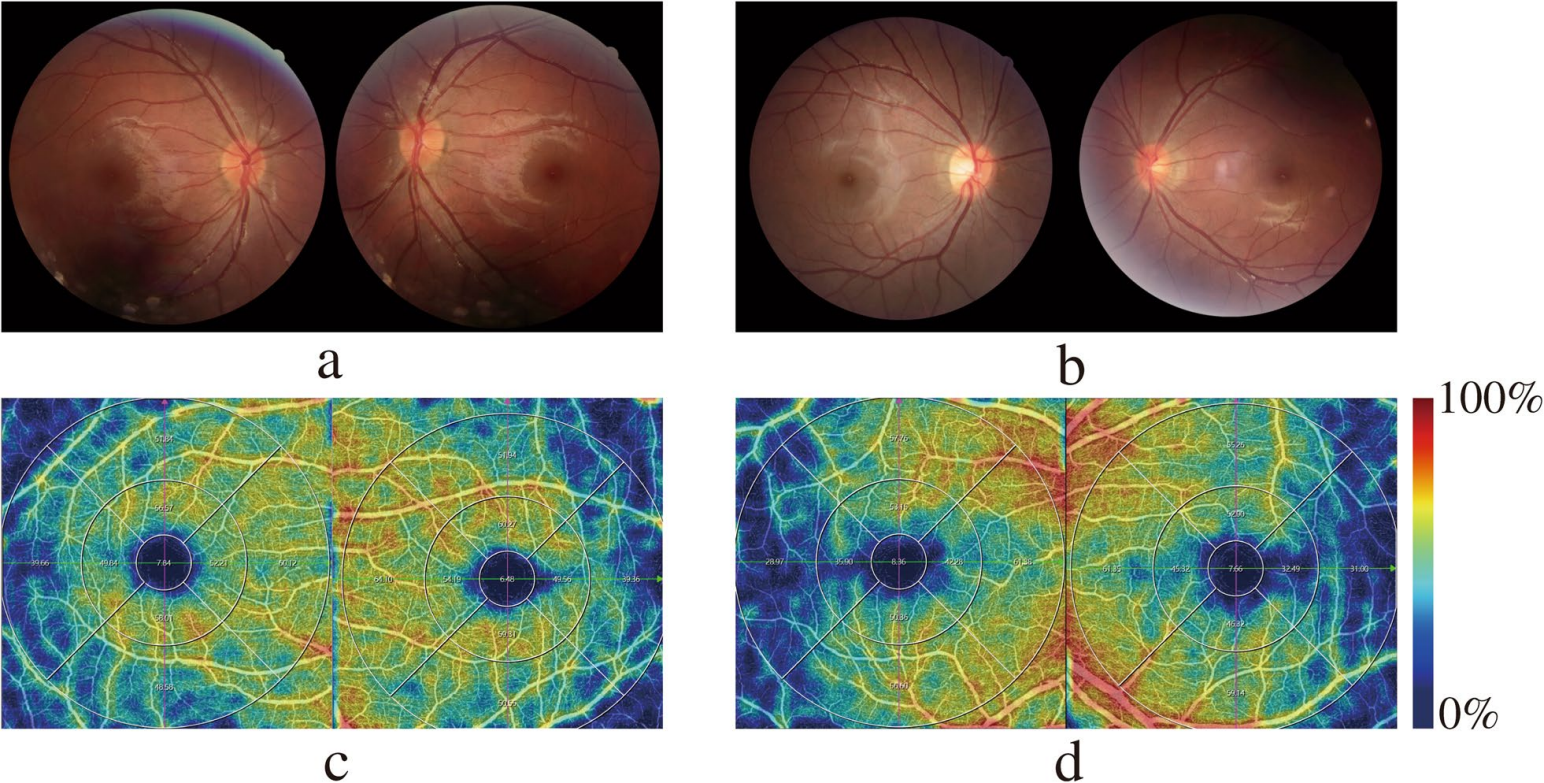
Fundus photography and retinal vascular density maps of both eyes of patients and healthy controls. **a** Fundus photography of both eyes of a patient. **b** Fundus photograph of a healthy control. **c** Vascular density map of the macular SVC of a patient. **d** Vascular density map of the macular SVC of a healthy control

OCTA imaging was performed by OCTA operators under dimmed-light conditions on one ophthalmic optical coherence tomography system (machine model VG100C, version 2.1.016; Henan Shiwei Imaging Technology Ltd., China), centred on the fovea and optic disc, and a 6*6 mm rectangle was scanned for 100,000 A-scans per second. The motion trajectories of erythrocytes were obtained through comparison with the decoherence algorithm, and three-dimensional reconstruction was subsequently performed to obtain each participant's macular and peripapillary microvascular and perfusion images. Macular and peripapillary partitioning and layering are automatically performed by the system (Fig. 1). With the fovea as the centre of the circle, the Early Treatment of Diabetic Retinopathy Study (ETDRS) grid was used to segment the macular (Fig. 1a, b), in which the diameter of the central circle was 1 mm, the diameter of the pericentral ring was 3 mm, and the diameter of the peripheral ring was 6 mm and was divided into four quadrants—Superior(S), Inferior(I), Nasal(N) and Tempo(T)—by two mutually perpendicular straight lines. The software automatically uses the ONH grid [4] to segment the peripapillary radial capillary network with the optic disc centre as the centre of the circle (Fig. 1c, d) and defines the peripapillary area as extending to a 1.0-mm-wide circular ring along a circle with a diameter of 2.0 mm in the optic disc while dividing the circular ring into eight sectors: nasal superior (NS), nasal inferior (NI), inferior nasal (IN), inferior tempo (IT), tempo inferior (TI), tempo superior (TS), superior tempo (ST) and superior nasal (SN). Since some participants had poor visual fixation, the positions of the centre of the macular and optic disc automatically located by the system may have been inaccurate, and these inaccurate positions were manually corrected. The scanning system automatically calculates data for each area, including the foveal avascular zone (FAZ) area, vascular density and perfusion area. Vascular density represents the percentage of the area occupied by the vasculature in the analysis area. The retinal capillary network is divided into the superficial vascular complex (SVC) and deep vascular complex (DVC). The SVC includes the inner limiting membrane to the inner one-third of the inner plexiform layer, which contains the superficial capillary network. The DVC includes the inner one-third of the inner plexiform layer to the junction of the inner nuclear layer and the outer plexiform layer, which contains a middle capillary network and a deep capillary network (Fig. 1e, f). Figure 2 shows the fundus photographs and macular SVC vascular density maps of both the eyes of a patient and a healthy control subject of the same age. The patient's macular SVC vascular density decreased.

### VLCFAs in plasma

The plasma sample information was provided by Southwest Hospital in Chongqing, China. The plasma samples were taken from the vein. Gas chromatography‒mass spectrometry was used to analyse and test the serum concentrations of C22:0, C24:0 and C26:0, after which we calculated the serum concentration ratios (C24:0/C22:0, C26:0/C22:0).

### Data analysis

All the data were statistically analysed via IBM SPSS Statistics 26. The data were tested for normality via the Shapiro‒Wilk test. First, the Kruskal‒Wallis test was used to evaluate whether there were differences in age among the three groups (symptomatic group, less symptomatic group and healthy control group). Second, we used generalized estimation equations to compare the FAZ area, retinal vascular density, and perfusion area in different sectors among the three groups. We then conducted pairwise comparisons to determine which groups were significantly different from the other groups. Finally, using the average of the results from each participant’s binoculus for analysis, we performed Spearman correlation analysis among C24:0/C22:0, C26:0/C22:0, the EDSS score, the SSPROM score, the retinal vascular density and the perfusion area.

The significance level for all the above statistical tests was 0.05 (α = 0.05, two-sided).

## Results

### Patient characteristics

**Fig. 3:**
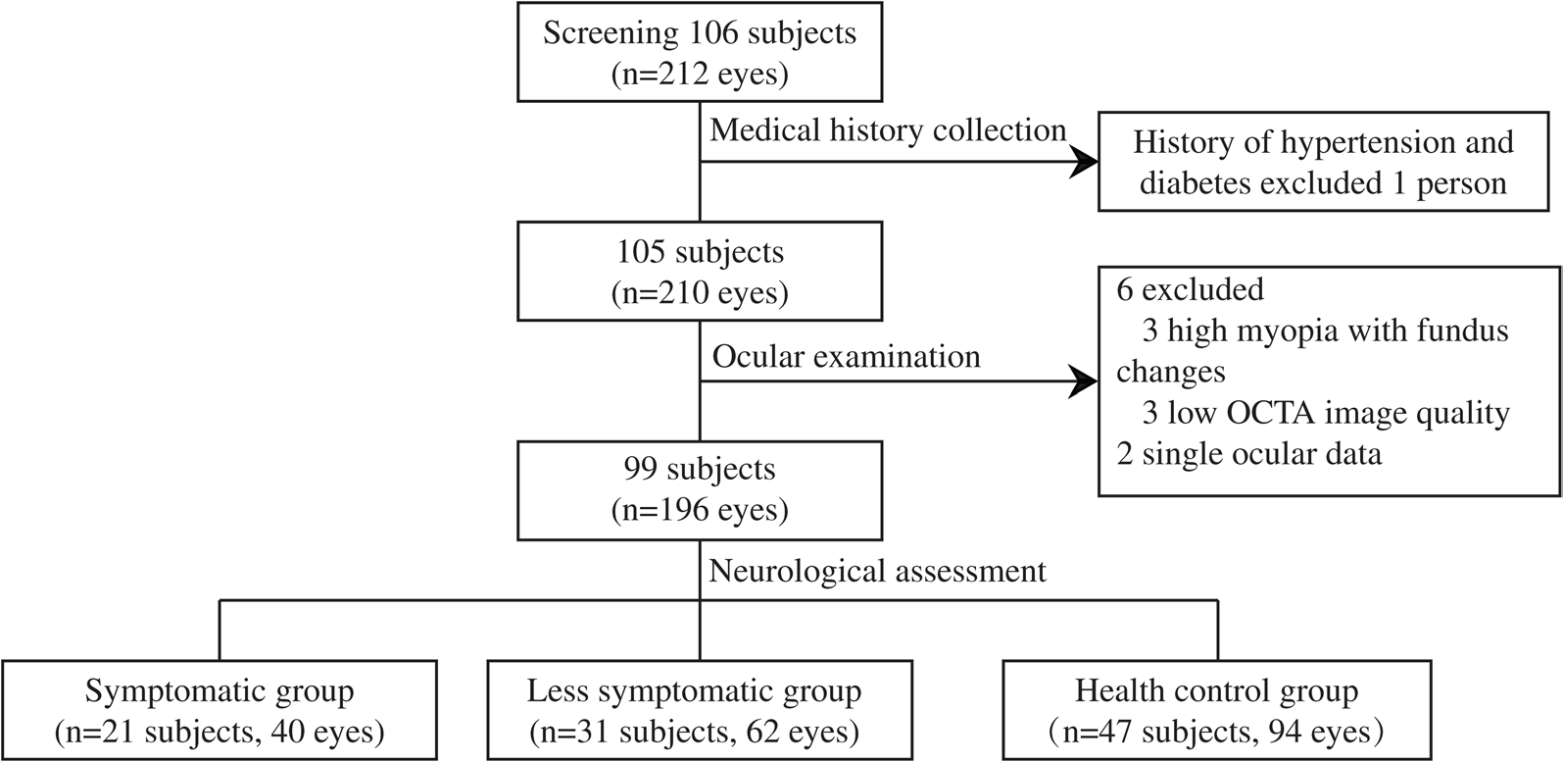
Subject enrolment and exclusions

A total of 106 participants were screened for this study. Seven participants were excluded because of low OCTA image quality (n = 3), a history of hypertension or diabetes (n = 1) or high myopia with fundus changes (n = 3). Finally, a total of 99 participants were included (52 patients and 47 healthy controls). One patient had monocular amblyopia, and only 1 patient had been collected only one eye; thus, a total of 196 eyes were included (Fig. 3).

Twenty-one of the 52 patients (40.38%) were classified into the symptomatic group; the median rating of the EDSS score was 4.0 (ranging from 1.5 to 7.5), and the median rating of the SSPROM score was 86.5 (ranging from 62.0 to 100.0). Thirty-one patients (59.62%) were classified into the less symptomatic group; the median rating of the EDSS score was 1.5 (ranging from 0 to 4.0), and the median rating of the SSPROM score was 99.0 (ranging from 89.0 to 100.0).

There was no statistically significant difference in the median age of the three groups of subjects (healthy control group vs. less symptomatic group vs. symptomatic group, 33 vs. 36 vs. 25, p = 0.146).

### Retinal vascular density and perfusion function

First, we compared the FAZ area among the three groups and found no significant difference (Wald chi-square = 4.978, p = 0.083) (Table 1).

**Table 1:** FAZ area comparison (mm^2^)

Classification	Healthy control group (n = 94)	Less symptomatic group (n = 62)	Symptomatic group (n = 40)	Wald Chi-Square	p
FAZ	0.359123 (0.326671–0.391576)	0.359124 (0.310912–0.407336)	0.301976 (0.259579–0.344373)	4.978	0.083

The data are expressed as the means (95% Wald confidence intervals)

**Fig. 4:**
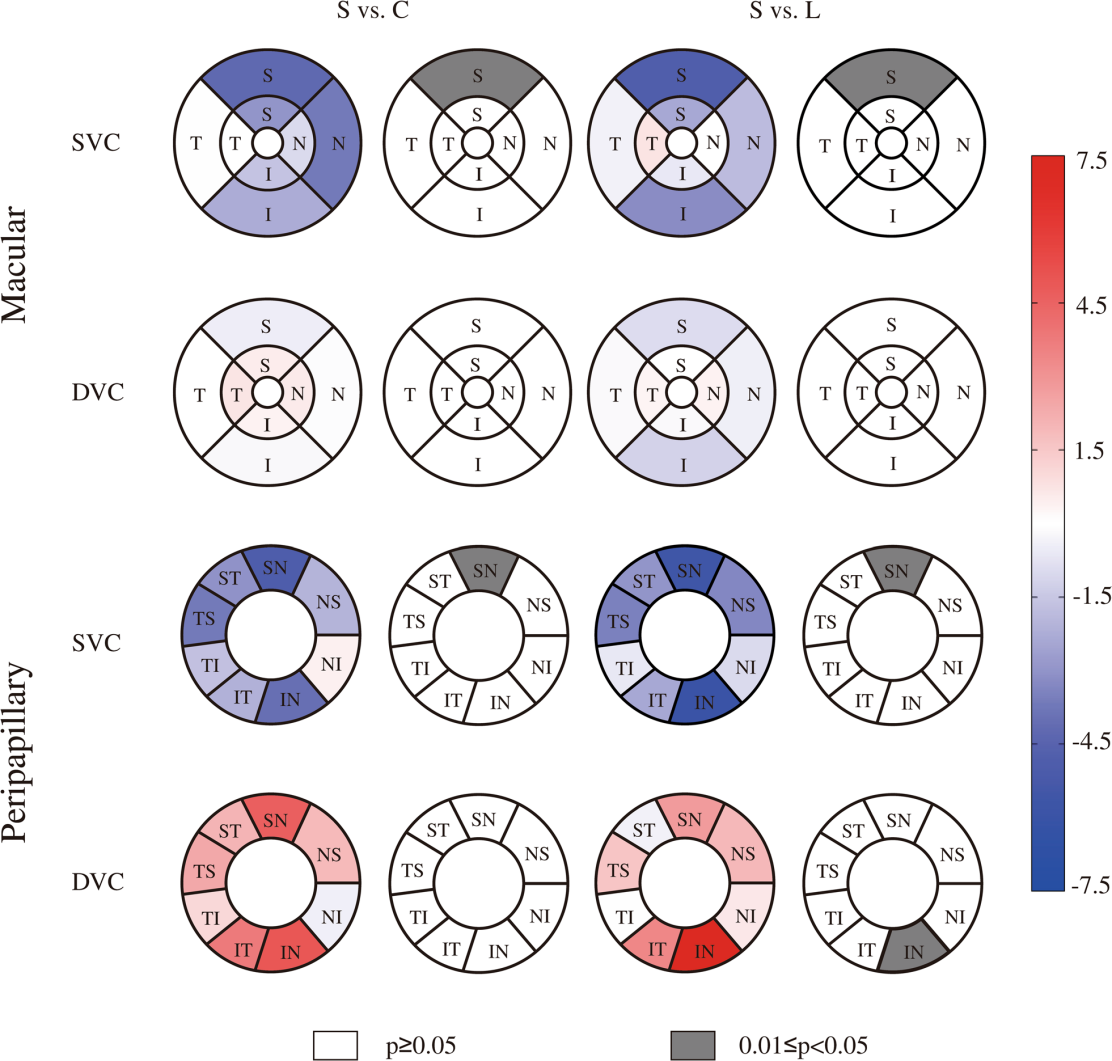
Comparative map and p value map of vascular density. The values in the sector are the differences in the means or medians between the symptomatic group and the other two groups. *S* symptomatic group, *C* healthy control group, *L* less symptomatic group

Second, we compared the differences in vascular density in different sectors of the superficial and deep vascular complexes of the macular and peripapillary region among the three groups (Table 2). There were significant differences among the three groups in the peripheral ring S quadrant of the macular SVC (Wald chi-square = 8.160, p = 0.017), the SN sector of the peripapillary SVC (Wald chi-square = 8.265, p = 0.016) and the IN sector of the peripapillary DVC (Wald chi-square = 7.336, p = 0.026). Compared with the healthy control group, the symptomatic group presented significantly lower vascular density in the peripheral ring S quadrant of the macular SVC (mean difference, − 4.259225; 95% CI − 8.248627  to  − 0.269823; p = 0.032) and in the SN sector of the peripapillary SVC (mean difference, − 4.940884; 95% CI − 9.655061  to − 0.226707; p = 0.036). Compared with the less symptomatic group, the symptomatic group had significantly lower vascular density in the peripheral ring S quadrant of the macular SVC (mean difference, − 4.817846; 95% CI − 8.924294  to  − 0.711399; p = 0.015) and in the SN sector of the peripapillary SVC (mean difference, − 5.635483; 95% CI − 10.450009  to  − 0.820957; p = 0.015) but greater vascular density in the IN sector of the peripapillary DVC (mean difference, 7.209401; 95% CI 0.818716–13.600086; p = 0.021). There was no statistically significant difference in the remaining sectors. No significant differences were found across all sectors between the less symptomatic group and the healthy control group. Figure 4 shows the comparative and p value maps of vascular density between the symptomatic group and the healthy control group and between the symptomatic group and the less symptomatic group.

**Table 2:** Comparison of the vascular density of the macular and peripapillary regions in different sectors (%)

Region	Layer	Sector	Healthy control group (n = 94)	Less symptomatic group (n = 62)	Symptomatic group (n = 40)	Wald Chi-square	p	p (pairwise comparisons)		
								L vs. C	S vs. C	S vs. L
Macular	Pericentral Ring SVC	S	54.281324 (53.074503–55.488146)	53.686327 (51.852345–55.520310)	51.266850 (48.055841–54.477860)	3.005	0.223			
		T	43.972240 (42.575828–45.368653)	43.242166 (41.672699–44.811634)	43.974670 (41.404428–46.544912)	0.517	0.772			
		I	54.470100 (52.998954–55.941246)	53.464719 (51.886200–55.043239)	52.836462 (50.093176–55.579748)	1.437	0.488			
		N	48.338259 (46.981180–49.695337)	47.313223 (45.359434–49.267011)	47.320205 (44.152005–50.488405)	0.872	0.646			
	Peripheral Ring SVC	S	51.364501 (49.986411–52.742591)	51.923123 (50.329983–53.516262)	47.105276 (44.144857–50.065695)	8.160	**0.017**	1.000	**0.032**	**0.015**
		T	34.932885 (33.512706–36.353064)	35.366119 (34.169651–36.562588)	34.965155 (32.805288–37.125023)	0.243	0.886			
		I	50.697633 (49.389476–52.005790)	51.577269 (49.950075–53.204464)	48.396714 (45.486460–51.306968)	3.510	0.173			
		N	61.465818 (60.326055–62.605581)	60.702755 (58.797956–62.607554)	57.330694 (53.173092–61.488296)	3.713	0.156			
	Pericentral Ring DVC	S	59.758285 (59.068610–60.447961)	60.231405 (59.245774–61.217036)	60.259604 (59.214929–61.304280)	0.918	0.632			
		T	58.741731 (57.912611–59.570851)	59.156687 (58.109661–60.203713)	59.430314 (58.454315–60.406314)	1.148	0.563			
		I	59.315662 (58.609743–60.021580)	59.871681 (58.948242–60.795119)	59.666453 (58.567995–60.764910)	0.933	0.627			
		N	58.522529 (57.765851–59.279207)	58.743892 (57.466083–60.021701)	59.094852 (57.989529–60.200175)	0.705	0.703			
	Peripheral Ring DVC	S	58.452806 (57.656616–59.248996)	58.906794 (57.952080–59.861507)	57.963299 (56.959788–58.966810)	1.711	0.425			
		T	61.340332 (60.652744–62.027920)	61.527106 (60.612082–62.442131)	61.325855 (60.300071–62.351640)	0.121	0.941			
		I	56.879331 (56.038895–57.719767)	57.897844 (56.848547–58.947140)	56.673060 (55.406528–57.939593)	2.893	0.235			
		N	57.986857 (57.230822–58.742893)	58.317756 (57.205656–59.429857)	57.852909 (56.743164–58.962654)	0.366	0.833			
Peripapillary	SVC	NS	63.888087 (61.787743–65.988431)	65.156755 (63.296139–67.017370)	61.818475 (58.125120–65.511830)	2.688	0.261			
		NI	55.635271 (53.336294–57.934248)	57.066187 (54.814684–59.317690)	56.040787 (51.765727–60.315847)	0.779	0.677			
		IN	74.978331 (72.984691–76.971970)	76.964952 (74.862155–79.067749)	70.965659 (66.383685–75.547632)	5.884	0.053			
		IT	84.990863 (83.782761–86.198964)	85.225973 (83.774866–86.677080)	82.787052 (79.660889–85.913214)	1.972	0.373			
		TI	68.262507 (66.329541–70.195474)	67.185890 (64.923926–69.447855)	66.527923 (62.348556–70.707291)	0.823	0.663			
		TS	68.236998 (66.486158–69.987838)	68.076213 (65.974280–70.178146)	64.565208 (59.976384–69.154032)	2.199	0.333			
		ST	82.017472 (80.606897–83.428048)	81.959173 (79.942691–83.975654)	78.953965 (75.552677–82.355252)	2.758	0.252			
		SN	77.802289 (75.899792–79.704787)	78.496889 (76.435514–80.558263)	72.861405 (69.500090–76.222720)	8.265	**0.016**	1.000	**0.036**	**0.015**
Peripapillary	DVC	NS	35.681567 (33.466439–37.896695)	35.622752 (34.121345–37.124158)	37.543064 (34.837029–40.249098)	1.572	0.456			
		NI	43.661186 (41.413345–45.909028)	42.613352 (40.539001–44.687703)	43.234005 (40.109765–46.358244)	0.457	0.796			
		IN	22.433369 (19.810059–25.056679)	20.148194 (17.453405–22.842982)	27.357594 (22.874287–31.840902)	7.336	**0.026**	0.701	0.189	**0.021**
		IT	15.659249 (13.672777–17.645721)	16.022908 (13.309437–18.736379)	19.403278 (15.511728–23.294827)	2.882	0.237			
		TI	47.288411 (45.443454–49.133367)	48.397776 (46.040669–50.754883)	48.335071 (45.129972–51.540170)	0.648	0.723			
		TS	46.717499 (44.826298–48.608700)	47.510584 (45.296577–49.724590)	49.118078 (46.798371–51.437785)	2.496	0.287			
		ST	20.194866 (17.808741–22.580990)	22.646105 (19.197710–26.094500)	22.241476 (19.083451–25.399500)	1.746	0.418			
		SN	18.672769 (16.143699–21.201840)	20.526671 (17.967245–23.086097)	23.290525 (19.826198–26.754852)	4.475	0.107			

1. The data are expressed as the means (95% Wald confidence intervals). The bold values indicate statistical significance

2. *C* Healthy control group, *L* less symptomatic group, *S* symptomatic group

**Fig. 5:**
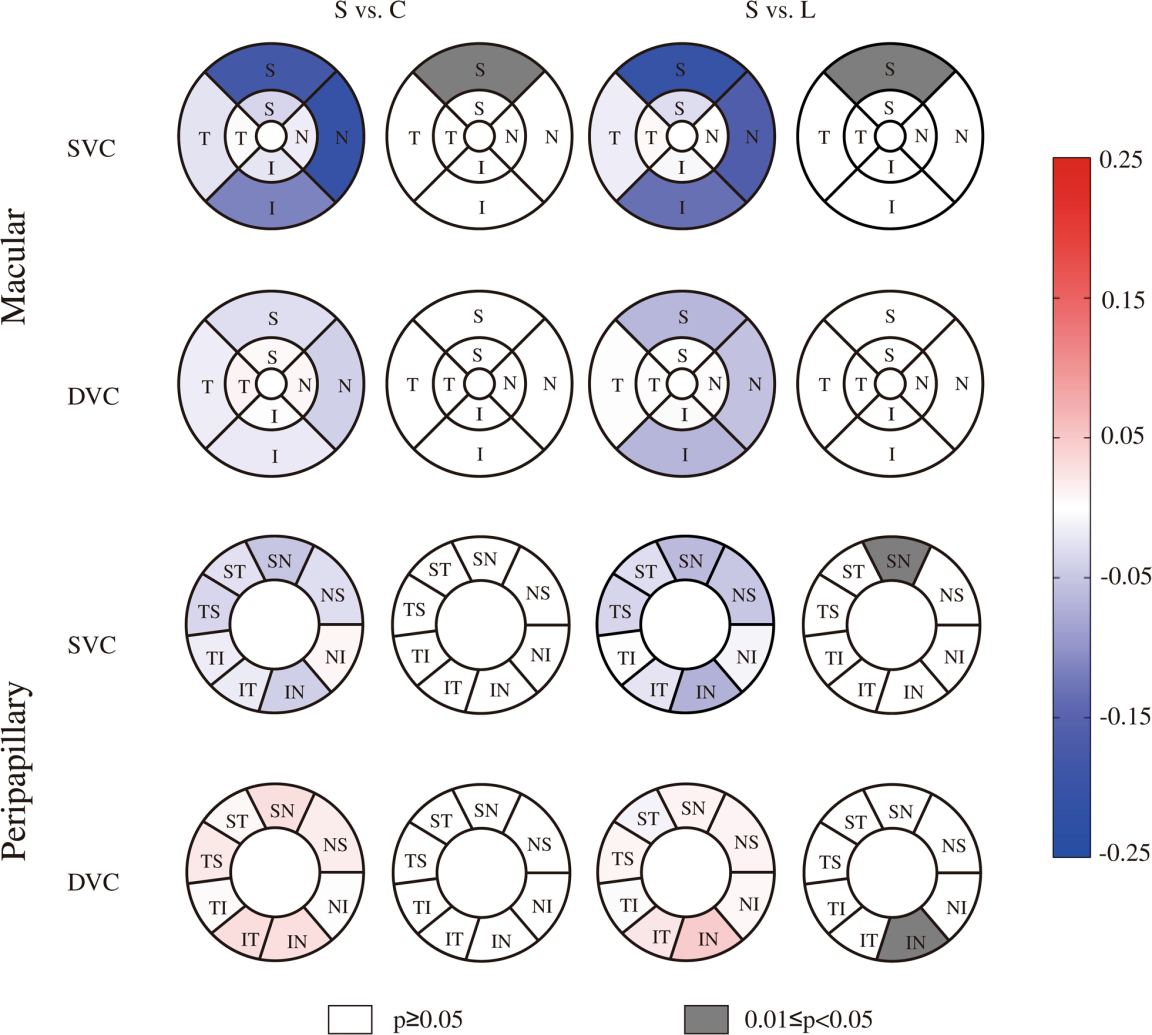
Comparative map and p value map of the perfusion area. The values in the sector are the differences in the means or medians between the symptomatic group and the other two groups. *S* symptomatic group, *C* healthy control group, *L* less symptomatic group

We then further compared the differences in perfusion area among the three groups in different sectors and layers of the macular and peripapillary region (Table 3). There were significant differences between the three groups in the peripheral ring S quadrant of the macular SVC (Wald chi-square = 9.242, p = 0.010), the SN sector of the peripapillary SVC (Wald chi-square = 8.556, p = 0.014) and the IN sector of the peripapillary DVC (Wald chi-square = 6.360, p = 0.042). Pairwise comparisons revealed that the symptomatic group had a significantly smaller perfusion area in the peripheral ring S quadrant of the macular SVC (mean difference, − 0.180304; 95% CI − 0.337135  to  − 0.023472; p = 0.018) compared with the healthy control group. Compared with the less symptomatic group, the symptomatic group had significantly less perfusion area in the peripheral ring S quadrant of the macular SVC (mean difference, − 0.202707; 95% CI − 0.369499  to  − 0.035915; p = 0.011) and SN sector of the peripapillary SVC (mean difference, − 0.063351; 95% CI − 0.116611  to  − 0.010091; p = 0.013) but more perfusion area in the IN sector of the peripapillary DVC (mean difference, 0.047320; 95% CI 0.001685–0.092956; p = 0.039). There were no statistically significant differences in the remaining sectors. No significant differences were found across all sectors between the less symptomatic group and the control group. Figure 5 shows the comparative map and p value map of the perfusion area between the symptomatic group and the healthy control group and between the symptomatic group and the less symptomatic group. Further analysis of quadrants with an interaction effect revealed that the vascular density (mean difference, 9.033783; 95% CI 3.105466–14.962099; p = 0.001) and perfusion area (mean difference, 0.056815; 95% CI 0.012426–0.101203; p = 0.007) in the oculus dexter SN sector of the peripapillary DVC in the symptomatic group were greater than those in the control group. However, no significant difference was detected in the oculus sinister (Supplementary Tables 1–2).

**Table 3:** Comparison of the perfusion areas of the macular and peripapillary regions in different sectors (mm^2^)

Region	Layer	Sector	Healthy control group (n = 94)	Less symptomatic group (n = 62)	Symptomatic group (n = 40)	Wald Chi-Square	p	p (pairwise comparisons)		
								L vs. C	S vs. C	S vs. L
Macular	Pericentral Ring SVC	S	0.789122 (0.772622–0.805622)	0.781582 (0.755205–0.807960)	0.750569 (0.709795–0.791342)	2.969	0.227			
		T	0.657550 (0.641451–0.673649)	0.646582 (0.626651–0.666513)	0.652285 (0.620594–0.683976)	0.707	0.702			
		I	0.795362 (0.775112–0.815612)	0.779798 (0.757389–0.802208)	0.772074 (0.735882–0.808265)	1.685	0.431			
		N	0.709040 (0.691074–0.727007)	0.694261 (0.668796–0.719727)	0.693070 (0.653404–0.732736)	1.126	0.570			
	Peripheral Ring SVC	S	2.554641 (2.495977–2.613306)	2.577045 (2.502013–2.652077)	2.374338 (2.260190–2.488486)	9.242	**0.010**	1.000	**0.018**	**0.011**
		T	1.903265 (1.850071–1.956459)	1.893645 (1.848007–1.939283)	1.877697 (1.791933–1.963461)	0.253	0.881			
		I	2.537223 (2.480297–2.594150)	2.555250 (2.478202–2.632298)	2.422042 (2.302383–2.541701)	3.594	0.166			
		N	2.969844 (2.909264–3.030423)	2.925360 (2.827699–3.023021)	2.763251 (2.577681–2.948822)	4.484	0.106			
	Pericentral Ring DVC	S	0.873891 (0.862336–0.885447)	0.880589 (0.864286–0.896891)	0.879834 (0.862579–0.897089)	0.567	0.753			
		T	0.848172 (0.835909–0.860436)	0.854395 (0.838088–0.870702)	0.857811 (0.842691–0.872931)	1.002	0.606			
		I	0.868136 (0.856493–0.879780)	0.875361 (0.860153–0.890570)	0.870517 (0.854125–0.886909)	0.548	0.761			
		N	0.849438 (0.837239–0.861638)	0.854973 (0.835011–0.874934)	0.857695 (0.838644–0.876746)	0.587	0.746			
	Peripheral Ring DVC	S	2.844040 (2.802705–2.885376)	2.880232 (2.834053–2.926411)	2.814037 (2.767951–2.860123)	3.965	0.138			
		T	2.940813 (2.898770–2.982856)	2.927429 (2.870790–2.984068)	2.924842 (2.861899–2.987786)	0.233	0.890			
		I	2.781810 (2.736399–2.827220)	2.829795 (2.770950–2.888640)	2.761747 (2.694046–2.829448)	2.544	0.280			
		N	2.860214 (2.819092–2.901336)	2.873161 (2.820494–2.925829)	2.817643 (2.748750–2.886536)	1.632	0.442			
Peripapillary	SVC	NS	1.023538 (0.990281–1.056796)	1.045016 (1.017660–1.072372)	0.993672 (0.941253–1.046091)	3.141	0.208			
		NI	0.716957 (0.689551–0.744364)	0.734105 (0.706370–0.761840)	0.724592 (0.676904–0.772279)	0.744	0.689			
		IN	0.904493 (0.876398–0.932587)	0.932276 (0.904554–0.959998)	0.860083 (0.805829–0.914337)	5.838	0.054			
		IT	0.807224 (0.789919–0.824530)	0.812674 (0.796776–0.828572)	0.788414 (0.756249–0.820579)	1.760	0.415			
		TI	0.659257 (0.639740–0.678775)	0.646711 (0.624196–0.669227)	0.643548 (0.604764–0.682332)	0.918	0.632			
		TS	0.764949 (0.744517–0.785381)	0.766269 (0.742319–0.790220)	0.727912 (0.680823–0.775001)	2.217	0.330			
		ST	0.767085 (0.748347–0.785823)	0.770485 (0.749272–0.791699)	0.740013 (0.706779–0.773247)	2.466	0.291			
		SN	0.877937 (0.852615–0.903259)	0.888406 (0.862999–0.913814)	0.825055 (0.789598–0.860513)	8.556	**0.014**	1.000	0.051	**0.013**
	DVC	NS	0.604935 (0.583616–0.626255)	0.610902 (0.597957–0.623846)	0.620660 (0.591691–0.649628)	0.739	0.691			
		NI	0.544581 (0.525964–0.563197)	0.534561 (0.517346–0.551777)	0.541958 (0.513215–0.570701)	0.629	0.730			
		IN	0.366365 (0.347975–0.384755)	0.348318 (0.329201–0.367435)	0.395638 (0.363552–0.427724)	6.360	**0.042**	0.547	0.361	**0.039**
		IT	0.250599 (0.236864–0.264334)	0.258974 (0.240138–0.277810)	0.280861 (0.260702–0.301019)	5.930	0.052			
		TI	0.456193 (0.443801–0.468584)	0.465871 (0.449264–0.482478)	0.461177 (0.438615–0.483739)	0.852	0.653			
		TS	0.522169 (0.506989–0.537350)	0.533842 (0.515931–0.551753)	0.542555 (0.523079–0.562031)	2.755	0.252			
		ST	0.279935 (0.265124–0.294746)	0.297455 (0.276427–0.318483)	0.286655 (0.268565–0.304746)	1.789	0.409			
		SN	0.316083 (0.298208–0.333958)	0.334124 (0.315867–0.352382)	0.344346 (0.321210–0.367483)	3.993	0.136			

1. The data are expressed as the means (95% Wald confidence intervals). The bold values indicate statistical significance

2*. C* Healthy control group, *L* less symptomatic group, *S* symptomatic group

### Correlations among retinal vascular density, perfusion function and disease severity

First, we analysed the correlations among patients’ C24:0/C22:0 and C26:0/C22:0 ratios and EDSS and SSPROM scores (Table 4). Among the 52 patients, there was a weak negative correlation between C26:0/C22:0 and SSPROM (ρ = − 0.364, p = 0.008) and a weak positive correlation between C26:0/C22:0 and EDSS (ρ = 0.398, p = 0.004). No obvious monotonic correlation between C24:0/C22:0 and EDSS or SSPROM was found. Second, the vascular density and perfusion area in the sectors of the patients’ retina that differed among the groups were correlated with the EDSS and SSPROM scores (Table 5). Among the 52 patients, the EDSS score was moderately negatively correlated with the vascular density (ρ = − 0.450, p = 0.001) and perfusion area (ρ = − 0.427, p = 0.002) of the peripheral ring S quadrant of the macular SVC and weakly negatively correlated with the vascular density in the SN sector of the peripapillary SVC (ρ = − 0.350, p = 0.011). No obvious monotonic correlation was found between the vascular density or perfusion area and the EDSS score in the other quadrants. No obvious monotonic correlation was found between SSPROM and retinal vascular density or perfusion area. We further analysed the correlation between vascular density and the perfusion area of ​​all quadrants and disease severity in the 52 patients (Supplementary Tables 3–6). We found that the EDSS score was correlated with vascular density in the peripheral ring I sector of the macular SVC (ρ = − 0.281, p = 0.044) and the ST sector of the peripapillary SVC (ρ = − 0.293, p = 0.035) and with the perfusion area in the peripheral ring I sector of the macular SVC (ρ = − 0.290, p = 0.037), the IT sector of the peripapillary DVC (ρ = 0.294, p = 0.034), the ST sector of the peripapillary SVC (ρ = − 0.332, p = 0.016), and the SN sector of the peripapillary SVC (ρ = − 0.356, p = 0.009). Third, correlation analysis was conducted between the vascular density and perfusion area of the above retinal sectors and between C24:0/C22:0 and C26:0/C22:0 (Table 6). Among the 52 patients, C26:0/C22:0 showed a weak negative correlation with vascular density (ρ = − 0.350, p = 0.011) and perfusion area (ρ = − 0.356, p = 0.010) in the IN sector of the peripapillary SVC. C26:0/C22:0 showed a weak positive correlation with the vascular density (ρ = 0.350, p = 0.028) and perfusion area (ρ = 0.288, p = 0.038) in the IN sector of the peripapillary DVC. No obvious monotonic correlation was found between the vascular density or perfusion area in other sectors and C26:0/C22:0. No obvious monotonic correlation between C24:0/C22:0 and the above sectors of the retina was found.

**Table 4:** Correlations between C24:0/C22:0 or C26:0/C22:0 and disease severity

	C24:0/C22:0	C26:0/C22:0
SSPROM (n = 52)		
Spearman’s Rho	− 0.202	− **0.364**
p	0.150	**0.008**
EDSS (n = 52)		
Spearman’s Rho	0.249	**0.398**
p	0.075	**0.004**

C22:0 is behenic acid, C24:0 is tetracosanoic acid, C26:0 is hexadecanoic acid, SSPROM is the severity scoring system for progressive myelopathy, and EDSS is the expanded disability status score. The bold values indicate statistical significance

**Table 5:** Correlations between retinal vascular density, perfusion area and disease severity

	Vascular density in peripheral ring S quadrant of macular SVC	Vascular density of peripapillary			Perfusion area in peripheral ring S quadrant of macular SVC	Perfusion area of peripapillary	
		IN of SVC	SN of SVC	IN of DVC		IN of SVC	IN of DVC
SSPROM (n = 52)							
Spearman’s Rho	0.261	0.046	0.257	− 0.189	0.267	0.043	− 0.273
p	0.062	0.743	0.066	0.180	0.056	0.765	0.051
EDSS (n = 52)							
Spearman’s Rho	− **0.450**	− 0.082	− **0.350**	0.124	− **0.427**	− 0.095	0.172
p	**0.001**	0.561	**0.011**	0.380	**0.002**	0.502	0.222

SSPROM is the severity scoring system for progressive myelopathy, and EDSS is the expanded disability status score. The bold values indicate statistical significance.

**Table 6:** Correlations between retinal vascular density, perfusion area, C24:0/C22:0 ratio and C26:0/C22:0 ratio

	Vascular density in peripheral ring S quadrant of macular SVC	Vascular density of peripapillary			Perfusion area in peripheral ring S quadrant of macular SVC	Perfusion area of peripapillary	
		IN of SVC	SN of SVC	IN Of DVC		IN of SVC	IN of DVC
C24:0/C22:0 (n = 52)							
Spearman’s Rho	− 0.018	− 0.141	− 0.223	0.124	0.062	− 0.162	0.106
p	0.901	0.318	0.112	0.381	0.663	0.252	0.455
C26:0/C22:0 (n = 52)							
Spearman’s Rho	− 0.156	− **0.350**	− 0.194	**0.305**	− 0.137	− **0.356**	**0.288**
p	0.270	**0.011**	0.168	**0.028**	0.335	**0.010**	**0.038**

C22:0 is behenic acid, C24:0 is tetracosanoic acid, and C26:0 is hexadecanoic acid. The bold values indicate statistical significance

## Discussion

This study revealed for the first time that XALD patients’ retinal vascular density and perfusion area were reduced in the SVC of the macular and the peripapillary region in partial quadrants but increased in the DVC. Moreover, changes in retinal vascular density and perfusion area are related to the severity of the disease and to changes in plasma VLCFA C26:0/C22:0.

In XALD patients with persistent inflammatory demyelination, the heterogeneity of capillary flow increases, and the blood‒brain barrier permeability of white matter around the lesion changes [19]. Degenerative lesions of the brain are closely related to ocular injury [22], and retinal microvascular injury is also associated with central nervous system disease, which has also been confirmed in other neurodegenerative diseases [15, 37, 41]. No previous studies have reported changes in retinal vascular density in patients with XALD. Our study revealed that the retinal vascular density in the peripheral ring S quadrant of the macular SVC and SN sector of the peripapillary SVC was significantly lower in the symptomatic group than in the healthy control group. Compared with that in the healthy control group, the perfusion area in the peripheral ring S quadrant of the macular SVC in the symptomatic group was significantly decreased. Compared with the less symptomatic group, the symptomatic group had significantly lower vascular density in the peripheral ring S quadrant of the macular SVC and SN sector of the peripapillary SVC and significantly less perfusion area in the peripheral ring S quadrant of the macular SVC and SN sector of the peripapillary SVC. Changes in retinal vascular density also occur in other neurodegenerative diseases [18]. Correlation analysis revealed that the EDSS score was moderately negatively correlated with the vascular density and perfusion area of the peripheral ring S quadrant of the macular SVC; weakly negatively correlated with the vascular density of the SN sector of the peripapillary SVC, the peripheral ring I sector of the macular SVC and the ST sector of the peripapillary SVC, the perfusion area in the peripheral ring I sector of the macular SVC, the ST sector of the peripapillary SVC, and the SN sector of the peripapillary SVC; and weakly positively correlated with the IT sector of the peripapillary DVC. This may indicate that as the disease severity in patients with XALD increases, the SVC vascular density and perfusion function decrease, whereas DVC increases. Larger cohort studies are needed to confirm this conclusion further.

Previous studies have suggested that the plasma level of VLCFAs is not related to the phenotype of XALD but may be related to genetic modifications and environmental factors [25]. Our study revealed that C26:0/C22:0 was weakly negatively correlated with the SSPROM score and weakly positively correlated with the EDSS score, which may be related to the low proportion of patients with severe CALD in our cohort. Moreover, we speculate that the plasma level of VLCFAs needs to accumulate over time to affect the severity of XALD. Asheuer and colleagues reported that there was a correlation between the accumulation of saturated VLCFAs in normal white matter and disease phenotype [1].

The neuropathology of the brains of patients with XALD involves many lipid-laden macrophages and a perivascular inflammatory response infiltrated by mononuclear cells and astrocytes in the lesion area [12]. Excessive VLCFAs in the plasma of patients with XALD promote the release of proinflammatory factors by initiating the proinflammatory response of macrophages. However, due to abnormalities in the ABCD1 gene in patients with XALD, macrophages are unable to increase peroxisome β-oxidation by upregulating the liver X receptor-mediated VLCFA transporter ABCD1. Therefore, the above proinflammatory response is prolonged and enhanced, reshaping the plasticity and invasiveness of macrophages and leading to a significant increase in phagocytosis by macrophages [49]. The accumulation of VLCFAs has a toxic effect on microglia, which results in their activation and subsequent apoptosis [10]. Abnormal lipids lead to neuronal PS exposure and upregulate the expression of MFGE8 and C1q through the TGFb1 pathway to increase the phagocytosis of microglia by exposed neurons [13]. We speculate that phagocytosis of activated microglia on neurons and inflammation impair cerebral microvascular endothelial cells [28] and the integrity of the blood‒brain barrier [20] in patients with XALD, leading to partial perfusion changes [29]. Our results show that C26:0/C22:0 has a weak negative correlation with the vascular density and perfusion area in the IN sector of the peripapillary SVC, which indicates that an increase in the proportion of VLCFAs in the plasma impairs the retinal microvasculature, resulting in a decrease in the vascular density and perfusion function of the retina SVC.

However, our study also revealed that the vascular density and perfusion area in the IN sector of the peripapillary DVC in the symptomatic group were significantly greater than those in the  less symptomatic group and that there was a weak positive correlation between C26:0/C22:0 and the vascular density and perfusion area in this sector. This may be due to the compensatory response to inflammatory stimulation [38], which leads to increases in the vascular density and perfusion area in the peripapillary DVC in patients with XALD.

In our study, changes in vascular density and perfusion area were found only in some quadrants of the retina. We speculate that this may be due to the route of retinal nerve fibres and the varying optic disc rim widths in different sectors or to the small sample size, which resulted in low test sensitivity. Our further analysis revealed that the vascular density and perfusion area in the oculus dexter SN sector of the peripapillary DVC in the symptomatic group were greater than those in the control group. However, no significant difference was found in the oculus sinister. Thus, the dynamic change process requires further cohort studies.

Studies have shown that the FAZ area changes in patients with cognitive impairment [7]. Cognitive impairment occurs in patients with CALD, but our study revealed no difference in the FAZ area between patients and healthy controls, which may be related to the small number of samples included in this study. Notably, patients with severe cognitive impairment were not included in this study because these patients could not cooperate to complete all the tests, which may also be one of the reasons why no difference in the area of the FAZ was found. Another shortcoming of this study is that SSPROM focuses more on evaluating the severity of myelopathy. This study uses two scale evaluation methods at the same time to reduce bias in the evaluation to some extent.

At present, the effective treatments for CALD patients are bone marrow transplantation and allogeneic haematopoietic stem cell transplantation, which need to be used before the disease progresses to brain demyelination [12]. Therefore, identifying a feasible and reliable biomarker for predicting and monitoring disease progression is necessary. Our study revealed changes in retinal vascular density and perfusion function in patients with XALD, which are related to the severity of the disease and the proportion of VLCFAs in the plasma. These results indicate that retinal vascular density and perfusion function have the potential to be used as biomarkers to monitor the progression of XALD. However, because of the slow progression of XALD, long-term follow-up is needed to elucidate the dynamic changes in the retinal vasculature in patients with XALD.

## Supplementary Information

Supplementary Material 1.

Supplementary Material 2.

## Data Availability

The datasets used and analyzed during the current study are available at http://www.medresman.org.cn/pub/cn/proj/projectshshow.aspx?proj=6066.

## Abbreviations

AMN: Adrenomyeloneuropathy
CALD: Cerebral ALD
DVC: Deep vascular complexes
EDSS: Expanded Disability Status Score
ETDRS: Early Treatment of Diabetic Retinopathy Study
FAZ: Fovea avascular zone
I: Inferior
IN: Inferior Nasal
IT: Inferior Tempo
N: Nasal
NI: Nasal Inferior
NS: Nasal Superior
OCTA: Optical coherence tomography angiography system
S: Superior
SN: Superior Nasal
SSPROM: Severity Scoring system for Progressive Myelopathy
ST: Superior Tempo
SVC: Superficial vascular complexes
T: Tempo
TI: Tempo Inferior
TS: Tempo Superior
VLCFAs: Very long-chain fatty acids
XALD: X-linked adrenoleukodystrophy
