# Supplementary material for: Optical coherence tomography angiography reveals abnormal retinal vascular density and perfusion in patients with X-linked adrenoleukodystrophy: a cross-sectional study
Source: Orphanet J Rare Dis. 2025 Jan 13;20:20. doi: 10.1186/s13023-024-03499-x (PMC11727801; doi:10.1186/s13023-024-03499-x)
Supplement: Supplementary file 1 — Supplementary Material 1. [file 13023_2024_3499_MOESM1_ESM.docx]

Supplementary table 1 Comparison of vascular density (%) and perfusion area (mm²) in oculus dexter SN sector of peripapillary DVC among three groups

|  | L vs C | S vs C | S vs L |
| --- | --- | --- | --- |
| Vascular Density | 4.050664(-0.966184~9.067512)  p=0.160 | 9.033783(3.105466~14.962099)  **p=0.001** | 4.983119(-0.833525~10.799762)  p=0.121 |
| Perfusion Area | 0.030952(-0.005378~0.067282)  p=0.124 | 0.056815(0.012426~0.101203)  **p=0.007** | 0.025863(-0.018629~0.070355)  p=0.492 |

1.Data are expressed as mean difference (95% Wald Confidence Interval for difference), n=97.

2*.C* health control group, *L* less symptomatic group, *S* symptomatic group.

Supplementary table 2 Comparison of vascular density (%) and perfusion area (mm²) in oculus sinister SN sector of peripapillary DVC among three groups

|  | L vs C | S vs C | S vs L |
| --- | --- | --- | --- |
| Vascular Density | -0.342860(-4.960796~4.275076)  p=1.000 | 0.201730(-5.922065~6.325524)  p=1.000 | 0.544590(-5.580515~6.669695)  p=1.000 |
| Perfusion Area | 0.005131(-0.028322~0.038583)  p=1.000 | -0.000288(-0.038491~0.037915)  p=1.000 | -0.005419(-0.044145~0.033307)  p=1.000 |

1.Data are expressed as mean difference (95% Wald Confidence Interval for difference), n=99

2*.C* health control group, *L* less symptomatic group, *S* symptomatic group.
