# Supplementary material for: Optical coherence tomography angiography reveals abnormal retinal vascular density and perfusion in patients with X-linked adrenoleukodystrophy: a cross-sectional study
Source: Orphanet J Rare Dis. 2025 Jan 13;20:20. doi: 10.1186/s13023-024-03499-x (PMC11727801; doi:10.1186/s13023-024-03499-x)
Supplement: Supplementary file 2 — Supplementary Material 2. [file 13023_2024_3499_MOESM2_ESM.docx]

Supplementary Table 3 Correlation between vascular density in all sectors of macular and disease severity

|  | DVC Of Pericentral Ring | | | |  | DVC Of Peripheral Ring | | | |  | SVC Of Pericentral Ring | | | |  | | SVC Of Peripheral Ring | | | |
| --- | --- | --- | --- | --- | --- | --- | --- | --- | --- | --- | --- | --- | --- | --- | --- | --- | --- | --- | --- | --- |
|  | S | T | I | N |  | S | T | I | N |  | S | T | I | N |  | S | | T | I | N |
| SSPROM(n=52) |  |  |  |  |  |  |  |  |  |  |  |  |  |  |  |  | |  |  |  |
| Spearman’s Rho | -0.116 | -0.020 | -0.040 | 0.020 |  | 0.141 | -0.055 | -0.041 | -0.021 |  | 0.118 | 0.104 | 0.086 | 0.105 |  | 0.261 | | -0.021 | 0.135 | 0.096 |
| p | 0.414 | 0.888 | 0.778 | 0.887 |  | 0.318 | 0.697 | 0.770 | 0.881 |  | 0.405 | 0.463 | 0.543 | 0.457 |  | 0.062 | | 0.885 | 0.339 | 0.500 |
| EDSS(n=52) |  |  |  |  |  |  |  |  |  |  |  |  |  |  |  |  | |  |  |  |
| Spearman’s Rho | 0.088 | 0.086 | 0.082 | 0.079 |  | -0.151 | -0.120 | -0.026 | -0.052 |  | -0.199 | -0.132 | -0.188 | -0.081 |  | **-0.450** | | -0.188 | **-0.281** | -0.230 |
| p | 0.534 | 0.545 | 0.561 | 0.579 |  | 0.285 | 0.396 | 0.853 | 0.715 |  | 0.158 | 0.351 | 0.183 | 0.569 |  | **0.001** | | 0.183 | **0.044** | 0.101 |

SSPROM is the Severity Scoring system for Progressive Myelopathy, EDSS is the Expanded Disability Status Score.

52 participants (n=52) included symptomatic group and less symptomatic group.

Supplementary Table 4 Correlation between perfusion area in all sectors of macular and disease severity

|  | DVC Of Pericentral Ring | | | |  | DVC Of Peripheral Ring | | | |  | SVC Of Pericentral Ring | | | |  | | SVC Of Peripheral Ring | | | |
| --- | --- | --- | --- | --- | --- | --- | --- | --- | --- | --- | --- | --- | --- | --- | --- | --- | --- | --- | --- | --- |
|  | S | T | I | N |  | S | T | I | N |  | S | T | I | N |  | S | | T | I | N |
| SSPROM(n=52) |  |  |  |  |  |  |  |  |  |  |  |  |  |  |  |  | |  |  |  |
| Spearman’s Rho | -0.090 | 0.002 | -0.017 | 0.036 |  | 0.234 | -0.091 | -0.062 | 0.030 |  | 0.145 | 0.130 | 0.121 | 0.116 |  | 0.267 | | -0.023 | 0.143 | 0.123 |
| p | 0.525 | 0.989 | 0.904 | 0.802 |  | 0.095 | 0.523 | 0.664 | 0.832 |  | 0.305 | 0.360 | 0.391 | 0.414 |  | 0.056 | | 0.872 | 0.312 | 0.385 |
| EDSS(n=52) |  |  |  |  |  |  |  |  |  |  |  |  |  |  |  |  | |  |  |  |
| Spearman’s Rho | 0.027 | 0.052 | 0.027 | 0.044 |  | -0.215 | 0.004 | -0.009 | -0.080 |  | -0.255 | -0.213 | -0.245 | -0.113 |  | **-0.427** | | -0.122 | **-0.290** | -0.228 |
| p | 0.848 | 0.712 | 0.852 | 0.755 |  | 0.125 | 0.975 | 0.952 | 0.573 |  | 0.068 | 0.129 | 0.080 | 0.425 |  | **0.002** | | 0.388 | **0.037** | 0.104 |

SSPROM is the Severity Scoring system for Progressive Myelopathy, EDSS is the Expanded Disability Status Score.

52 participants (n=52) included symptomatic group and less symptomatic group.

Supplementary Table 5 Correlation between vascular density in all sectors of peripapillary and disease severity

|  |  | DVC | | | | | | | |  | SVC | | | | | | | | |
| --- | --- | --- | --- | --- | --- | --- | --- | --- | --- | --- | --- | --- | --- | --- | --- | --- | --- | --- | --- |
|  |  | NS | NI | IN | IT | TI | TS | ST | SN |  | NS | NI | IN | IT | TI | TS | ST | SN |  |
| SSPROM(n=52) |  |  |  |  |  |  |  |  |  |  |  |  |  |  |  |  |  |  |  |
| Spearman’s Rho |  | -0.222 | -0.074 | -0.189 | -0.149 | -0.086 | -0.179 | 0.018 | -0.155 |  | 0.123 | -0.046 | 0.046 | -0.203 | -0.156 | -0.007 | 0.026 | 0.257 |  |
| p |  | 0.114 | 0.601 | 0.180 | 0.291 | 0.546 | 0.204 | 0.899 | 0.272 |  | 0.384 | 0.745 | 0.743 | 0.149 | 0.268 | 0.962 | 0.856 | 0.066 |  |
| EDSS(n=52) |  |  |  |  |  |  |  |  |  |  |  |  |  |  |  |  |  |  |  |
| Spearman’s Rho |  | 0.035 | -0.085 | 0.124 | 0.257 | 0.019 | 0.258 | 0.161 | 0.219 |  | -0.177 | -0.066 | -0.082 | -0.082 | -0.052 | -0.183 | **-0.293** | **-0.350** |  |
| p |  | 0.804 | 0.548 | 0.380 | 0.066 | 0.892 | 0.065 | 0.256 | 0.119 |  | 0.210 | 0.641 | 0.561 | 0.564 | 0.716 | 0.195 | **0.035** | **0.011** |  |

SSPROM is the Severity Scoring system for Progressive Myelopathy, EDSS is the Expanded Disability Status Score.

52 participants (n=52) included symptomatic group and less symptomatic group.

Supplementary Table 6 Correlation between perfusion area in all sectors of peripapillary and disease severity

|  |  | DVC | | | | | | | |  | SVC | | | | | | | | |
| --- | --- | --- | --- | --- | --- | --- | --- | --- | --- | --- | --- | --- | --- | --- | --- | --- | --- | --- | --- |
|  |  | NS | NI | IN | IT | TI | TS | ST | SN |  | NS | NI | IN | IT | TI | TS | ST | SN |  |
| SSPROM(n=52) |  |  |  |  |  |  |  |  |  |  |  |  |  |  |  |  |  |  |  |
| Spearman’s Rho |  | -0.168 | -0.098 | -0.273 | -0.236 | -0.083 | -0.193 | -0.009 | -0.127 |  | 0.135 | 0.002 | 0.043 | -0.201 | -0.175 | 0.025 | 0.038 | 0.254 |  |
| p |  | 0.233 | 0.488 | 0.051 | 0.092 | 0.556 | 0.171 | 0.949 | 0.370 |  | 0.340 | 0.991 | 0.765 | 0.154 | 0.215 | 0.858 | 0.791 | 0.069 |  |
| EDSS(n=52) |  |  |  |  |  |  |  |  |  |  |  |  |  |  |  |  |  |  |  |
| Spearman’s Rho |  | -0.080 | -0.132 | 0.172 | **0.294** | -0.066 | 0.197 | 0.156 | 0.112 |  | -0.206 | -0.132 | -0.095 | -0.112 | -0.067 | -0.254 | **-0.332** | **-0.356** |  |
| p |  | 0.571 | 0.350 | 0.222 | **0.034** | 0.642 | 0.162 | 0.270 | 0.429 |  | 0.142 | 0.350 | 0.502 | 0.431 | 0.638 | 0.069 | **0.016** | **0.009** |  |

SSPROM is the Severity Scoring system for Progressive Myelopathy, EDSS is the Expanded Disability Status Score.

52 participants (n=52) included symptomatic group and less symptomatic group.
